# The ‘lactate paradox’ revisited after 35 years: *In memoriam* Paolo Cerretelli

**DOI:** 10.1113/EP094234

**Published:** 2026-09-28

**Authors:** Bruno Grassi

**Affiliations:** ^1^ Department of Medicine University of Udine Udine Italy

**Keywords:** acclimatization, altitude, hypoxia, lactate metabolism

## Abstract

Some decades ago significant scientific interest was raised by the concept of the ‘lactate paradox’ occurring during altitude acclimatization. In short, in acute hypoxia blood lactate^−^ concentration ([La]_b_) is higher, compared to normoxia, at any given submaximal work rate, whereas peak [La]_b_ at exhausting exercise is unchanged. During altitude acclimatization the [La]_b_ vs. submaximal work rate relationship falls towards the original sea‐level curve, whereas peak [La]_b_ progressively falls with increasing altitude. Upon the premise that lactate^−^ accumulation derives from lack of O_2_ and anaerobic glycolysis, the lowered [La]_b_ at altitude was considered ‘paradoxical’. After decades of research on the ‘lactate shuttle’ and lactate metabolism the issue should be revisited. A new scenario could be summarized as follows. Muscle and blood lactate^−^ accumulation during increasing intensity exercise reflect a mismatch between a faster ‘speed’ of glycolysis and a relatively slower speed of oxidative phosphorylation, possibly aimed at accumulating lactate^−^ in blood, to be distributed for different purposes throughout the body. Acute hypoxia further accelerates glycolysis through an enhanced sympatho‐adrenergic stimulation, worsens intracellular hypoxia and increases [ADP]∙[P_i_]/[ATP], resulting in a greater [La]_b_ for the same absolute work rate. During altitude acclimatization the sympatho‐adrenergic stimulation falls, intracellular hypoxia is attenuated (mainly through a reduced O_2_ demand), the [ADP]∙[P_i_]/[ATP] increase subsides, and a tighter coupling between lactate^−^ production and utilization ensues, with the end result of decreased [La]_b_ for the same absolute work rate and lower peak [La]_b_. Although the perspective is radically changed, this remarkable series of metabolic adaptations appears of interest also today.

## Abstract (highlights)

- **What is the topic of this review?**
  Some decades ago a significant interest was raised by the concept of the ‘lactate paradox’, that arises during altitude acclimatization, in which peak blood lactate^−^ concentration ([La]_b_) following exercise progressively falls with increasing altitude.
- **What advances does it highlight?**
  After years of research on the “lactate shuttle”, a revisitation of the concept of the lactate paradox was needed. Mechanisms occurring during acclimatization lead to tighter coupling between lactate^−^ production and utilization, with the end result of decreased [La]_b_ for the same workrate and lower peak [La]_b_.

## 1 INTRODUCTION

Professor Paolo Cerretelli (Figure 1) died in Geneva, Switzerland, on 20 February 2026, at 93 years of age, 6 days after the death of his wife, Maria Grazia. For more than 50 years he had been one of the most influential researchers in exercise physiology, pulmonary gas exchange, skeletal muscle bioenergetics and proteomics, and altitude and environmental physiology. He had been Professor of Physiology in the medical schools of the Universities of Milano (Italy) and Geneva, and Director of research institutes of the National Research Council in Milano. He started his career under the guidance of Prof. Rodolfo Margaria at the Istituto di Fisiologia of the University of Milano, being for decades one of the leading figures of the ‘School of Margaria’. He spent long periods of research at the State University of New York at Buffalo and at the Max Planck Institute for Experimental Medicine in Göttingen (Germany). His intellectual curiosity, his extraordinary and contagious enthusiasm for research, his scientific rigor, and his continuous search for new topics of research are remembered and are a source of inspiration for dozens of his ‘pupils’ and physiologist colleagues around the world. I had the privilege to work under his guidance for several years at the beginning of my career, and the present paper, on a topic which was particularly ‘close to his heart’, is dedicated with gratitude to his memory.

**FIGURE 1:**
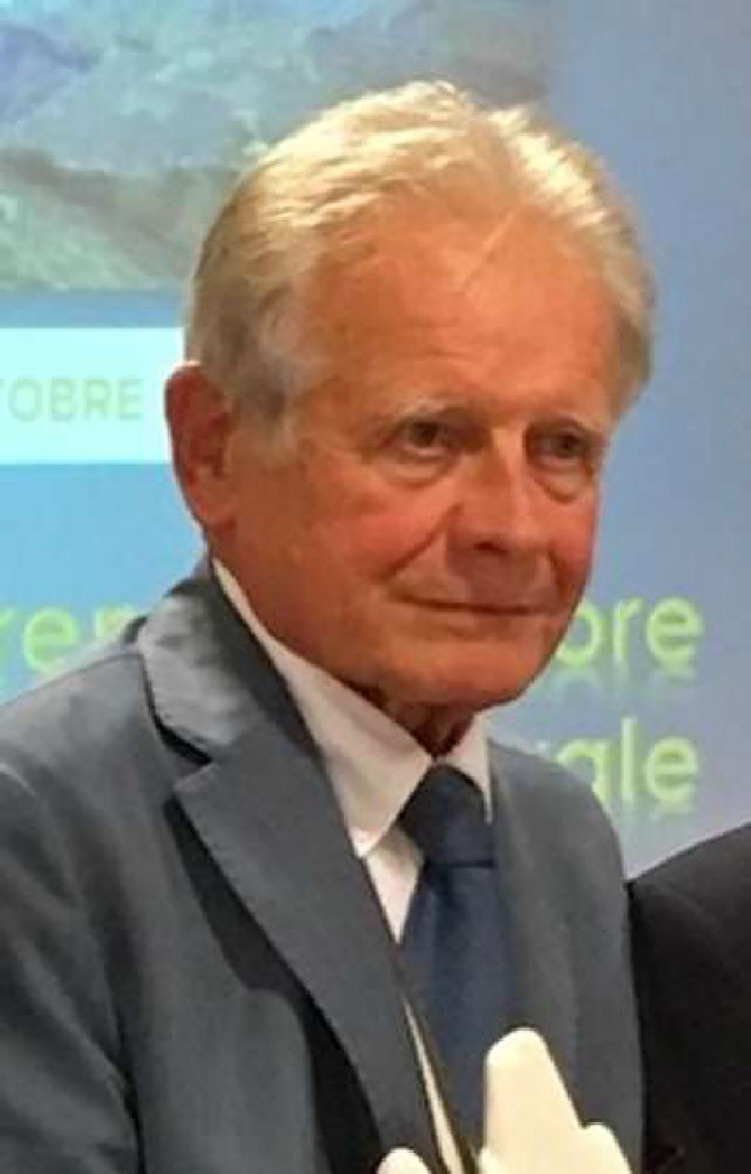
Prof. Paolo Cerretelli (1932–2026).

## 2 THE ‘LACTATE PARADOX’

In the late 1980s and early 1990s significant scientific interest was raised by the concept of the ‘lactate paradox’ occurring during altitude acclimatization (see, for example, the reviews by Cerretelli & Hoppeler, 1996; Cerretelli & Samaja, 2003; Cerretelli et al., 1982; Kayser, 1996; Reeves et al., 1992; Gladden, 1996; West, 1986). In short, in acute hypoxia blood lactate concentration ([La]_b_) is higher, compared to normoxia, at any given submaximal work rate, whereas peak [La]_b_ determined at exhausting exercise is unchanged. During altitude acclimatization the [La]_b_ vs. submaximal work rate exponential relationship falls, towards the original sea‐level curve, whereas peak [La]_b_ progressively falls with increasing altitude. A typical example of the lactate paradox is given in Figure 2 (data obtained at sea level and at an altitude of 5050 m, taken from Grassi et al. (1995, 1996)). If the peak [La]_b_ vs. altitude relationship is extrapolated to an altitude higher than about 7500 m, peak [La]_b_ would not be higher than [La]_b_ determined at rest (Cerretelli et al., 1982; West, 1986). Upon the concept that [La]_b_ is related to tissue hypoxia and anaerobic glycolysis (a concept which was challenged by George Brooks's ‘lactate shuttle’ paradigm and by several other authors, see below), this observation was defined a ‘paradox’ by John West (1986), since ‘it is indeed a paradox, because such a climber is apparently more hypoxic during maximal exercise than in any other known situation’. The term ‘lactate paradox’ was first utilized by Peter Hochachka a few years later (Hochachka, 1988). Also, the decreased [La]_b_ at any submaximal work rate was considered paradoxical by Reeves et al. (1992), upon the premises that convective O_2_ delivery to exercising muscle did not increase during acclimatization.

**FIGURE 2:**
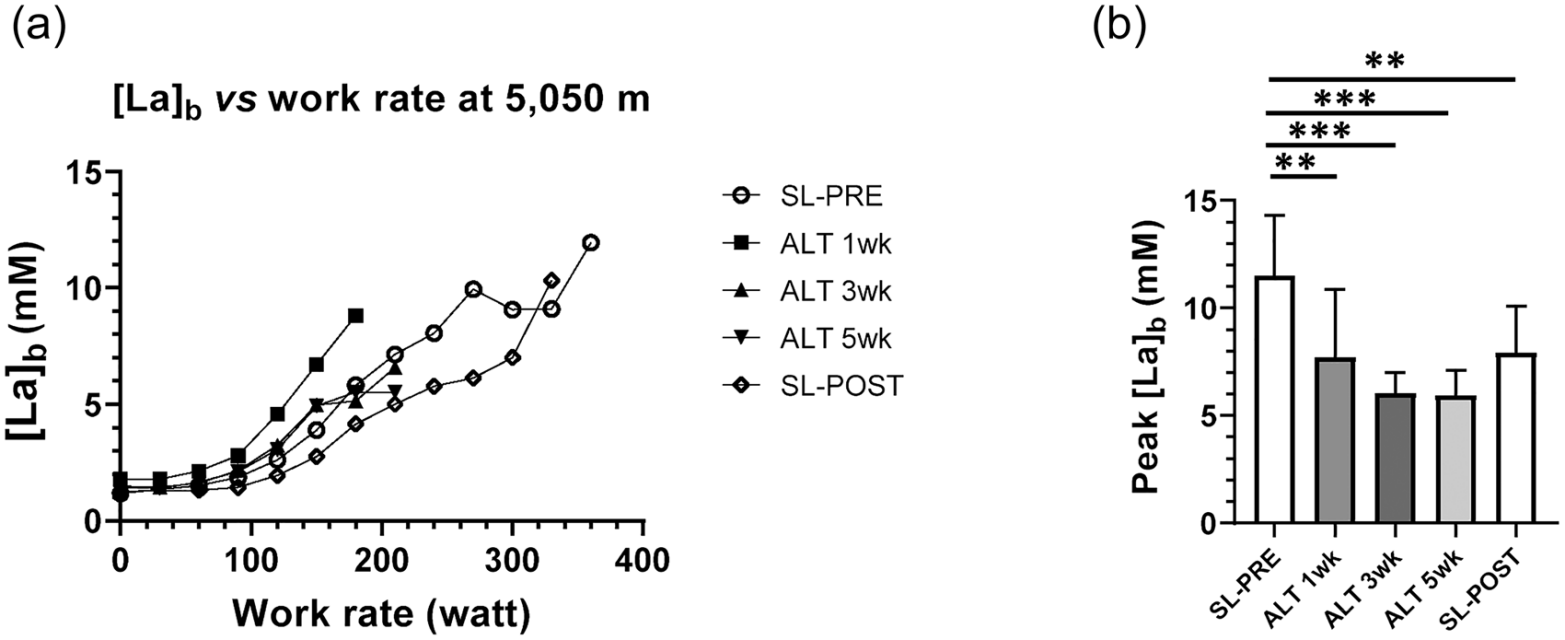
A typical example of the ‘lactate paradox’. (a) Blood lactate ([La]_b_) vs. work rate data, obtained at sea level before the expedition (SL‐PRE) and after 1, 3 and 5 weeks at 5050 m (ALT 1wk, ALT 3wk, ALT 5wk). The [La]_b_ vs. work rate relationship is shifted to the left (higher [La]_b_ for the same absolute work rate) at ALT 1wk vs. SL‐PRE, and shifts back towards SL‐PRE at ALT 3wk and ALT 5wk. (b) Peak [La]_b_ values (determined at exhaustion during an incremental cycle ergometer exercise) are lower at altitude vs. pre‐expedition, and are still lower vs. pre‐expedition 1 week after the return to sea level (SL‐POST). Data taken from Grassi et al. (1995, 1996). See text for further details. **P < 0.001, ***P < 0.001.

The interest in the lactate paradox was heightened a few years later by a series of studies carried out by Bengt Saltin's group, during scientific expeditions carried out mainly in the Andes (see, for example, Lundby & van Hall, 2002; Lundby et al., 2006; Van Hall et al., 2001; van Hall et al., 2009). In short, according to these researchers, in adequately motivated subjects, fully acclimatized to altitude, the lactate paradox does not exist, and lactate metabolism during exercise is not significantly different from that observed at sea level. van Hall et al. (2009), in particular, performed a comprehensive study including, among others, arterial, venous and muscle [lactate^−^] measurements, limb blood flow and other metabolic measurements on skeletal muscle biopsies. According to these authors changes in femoral venous [lactate^−^] at altitude would be simply dictated by changes in exercise intensity (van Hall et al., 2009). The controversy between the supporters and the detractors of the lactate paradox raised a lively and interesting debate (see, for example, Wagner & Lundby, 2007; West & van Hall, 2007), which I do not intend to re‐discuss here. Overall, it could be concluded that with some notable exceptions (Lundby & van Hall, 2002; Lundby et al., 2006; Van Hall et al., 2001; van Hall et al., 2009) most of the studies carried out by different groups on the topic did observe the lactate paradox, occurring in both acclimatized lowlanders and high altitude residents (Bender et al., 1989; Cerretelli, 1976; Edwards, 1936; Grassi et al., 1995; Grassi et al., 1996; Grassi et al., 2001; Hochachka, 1988; Hochachka et al., 1991; Kayser et al., 1994; Kayser, Ferretti et al., 1993; Pronk et al., 2003; Reeves et al., 1992; Sutton et al., 1988).

## 3 PAOLO CERRETELLI'S ROLE IN ALTITUDE PHYSIOLOGY AND IN THE CONCEPT OF THE LACTATE PARADOX

Paolo Cerretelli's contribution to altitude physiology has been impressive, spanning over 50 years, and including his participation as the scientific leader of expeditions to the Karakoram, Mt. Lothse Shar and Mt. Everest. His main interests were the limitations posed by altitude exposure on aerobic metabolism, the effects of acclimatization, the physiological characterization of elite altitude climbers, skeletal muscle adaptations, and during his last years of active research, the proteomic responses to hypoxia in skeletal muscle (see the reviews of Cerretelli, 1980; Cerretelli, 2013; Cerretelli & Hoppeler, 1996; Cerretelli et al., 1982; Gelfi et al., 2004). In all these topics specific attention was devoted to the adaptations in altitude populations, namely Sherpas and Tibetans.

Paolo Cerretelli was also one of the ‘fathers’ of the concept of the lactate paradox (Cerretelli & Hoppeler, 1996; Cerretelli & Samaja, 2003; Cerretelli et al., 1982), and its contribution to research in the field was extensive and original, mainly by work performed by his group in the ‘Pyramid’ Laboratory (Grassi et al., 1995; Grassi et al., 1996; Grassi et al., 2001; Kayser et al., 1994; Kayser, Ferretti et al., 1993; Kayser, Narici et al., 1993), established in 1990 by the Italian Ev‐K2‐CNR organization (mainly funded by the National Research Council of Italy) at 5050 m in the Khumbu valley (Nepal), not far from Mount Everest base camp (see Figure 3). The Pyramid expeditions were performed in close collaboration with the Royal Nepal Academy of Science and Technology (RONAST). Paolo Cerretelli, together with Prof. Ardito Desio, Mr Agostino Da Polenza and Dr Claudio Marconi, was instrumental in the establishment of the Pyramid Laboratory, and Cerretelli was the scientific leader of those early expeditions.

**FIGURE 3:**
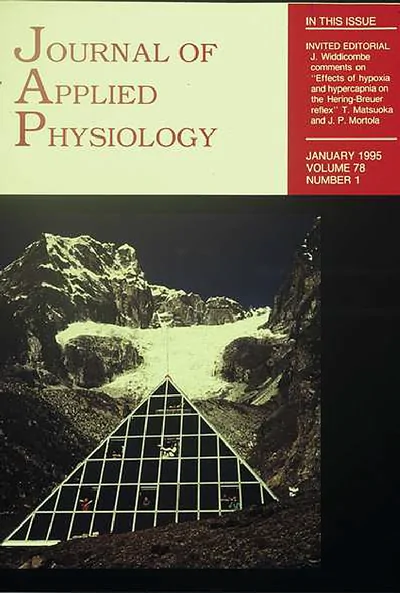
A photograph of the Pyramid Laboratory was published on the front cover of the *Journal of Applied Physiology* (volume 78, 1995).

The author of the present manuscript had the privilege to participate to some of these expeditions. The data from those studies are of interest also today, after about 35 years, although their interpretation needs to be at least in part revisited (see below). The Pyramid Laboratory, still active today, represented at the time a unique opportunity to perform altitude research, since it offered a real laboratory (see Figure 4), with adequate space, availability of instruments, electricity, together with real bedrooms and bathrooms, heating, a kitchen, a cook and a dining facility, full access to drinking water and Italian pasta, olive oil and parmesan cheese.

**FIGURE 4:**
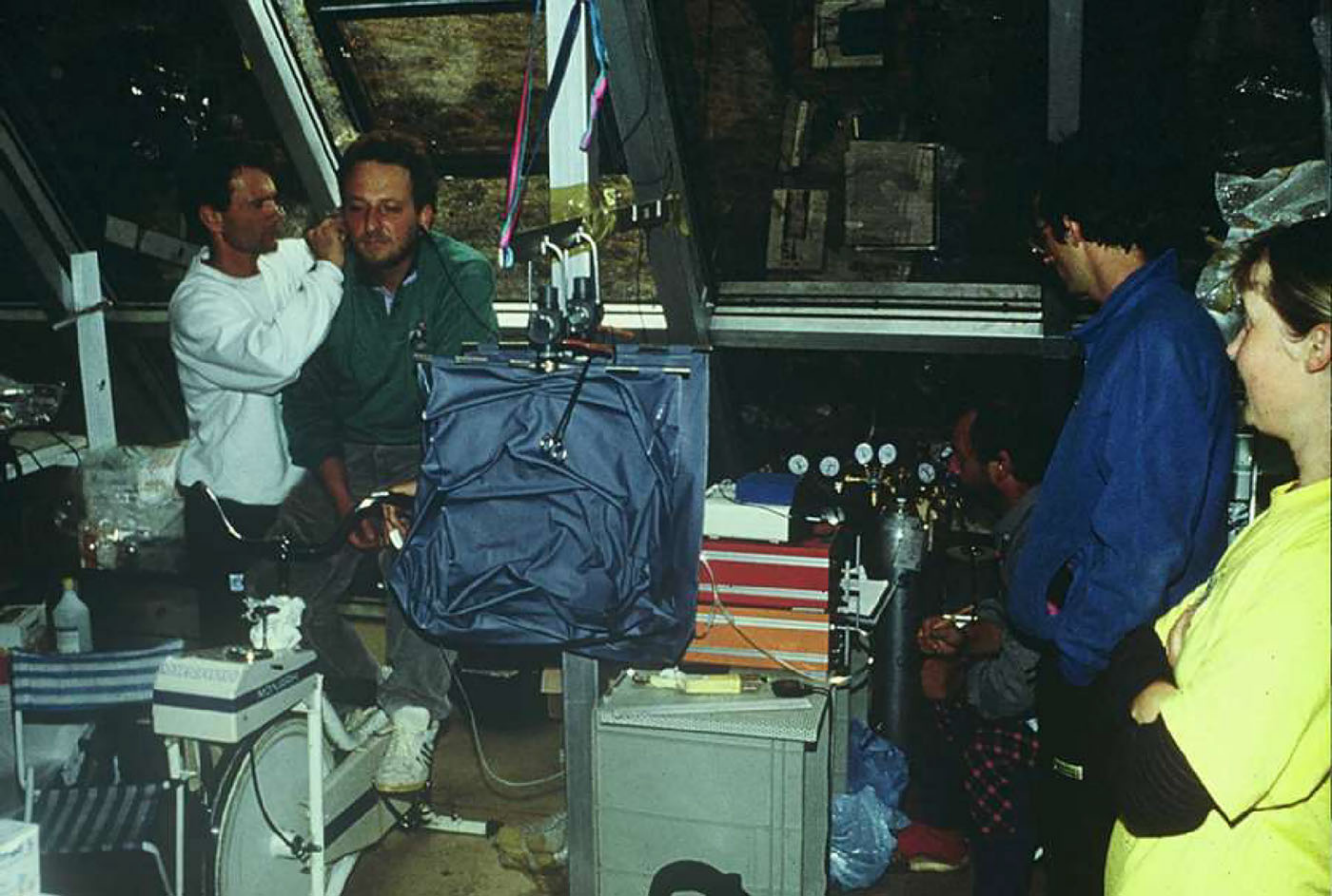
A photograph of one of the laboratories inside the Pyramid.

## 4 STUDIES PERFORMED IN THE PYRAMID LABORATORY

In the Pyramid, comfortable living conditions, full availability of drinking water and palatable food substantially prevented the significant muscle mass loss which is often observed at altitude (Cerretelli & Hoppeler, 1996; Hoppeler et al., 1990). The observed ∼4% decrease in body mass (vs. pre‐expedition) was likely attributable to changes in body water homeostasis, since the percentage of body fat (determined by skinfolds), the muscle+bone cross sectional area and the muscle+bone volume (determined in the leg by anthropometric measurements) were not significantly affected at altitude (Kayser, Narici et al., 1993). Also, the maximal voluntary force of the elbow flexors and the maximal jumping height (force platform) were not affected, pointing to the absence of muscle deterioration in terms of strength and ‘anaerobic alactic’ performance (Kayser, Narici et al., 1993). This concept was confirmed in another study performed after a few years (Grassi et al., 2001), in which no decreases in the average power output and peak [La]_b_ were observed during 10 s ‘all‐out’ exercise on a cycle ergometer.

On the other hand, in the Pyramid we definitively saw a ‘lactate paradox’. In some of these studies (Grassi et al., 1995; Grassi et al., 1996), measurements were carried out on 10 Caucasians staying for 5 weeks at 5050 m (Figure 2). At the end of a cycle ergometer incremental exercise, leading to exhaustion in ∼20 min, peak [La]_b_ (arterialized capillary blood samples) was lower after 1 week (7.7 ± 3.2 mM, mean ± SD), 3 weeks (6.0 ± 1.0 mM) and 5 weeks (6.0 ± 1.2 mM) at altitude, vs. 11.5 ± 2.8 mM at sea level before the expedition. Interestingly, peak [La]_b_ was still lower vs. pre‐expedition values for the first 2 weeks after the return from the expedition, in the presence of peak work rate and peak ${\dot{V}}_{O_{2}}$ values which were not significantly lower than those pre‐expedition (Grassi et al., 1996). Moreover, induction of acute hypobaric normoxia ($F_{IO_{2}}$ ∼0.40) at altitude increased peak work rate, whereas it induced only a relatively modest and non‐significant increase in peak [La]_b_ (Grassi et al., 1996), confirming that the decrease of this variable at altitude was not exclusively caused by the lower peak work rate, and was also not exclusively related to hypoxia. In other experiments lower peak [La]_b_ values (vs. pre‐expedition) were observed during exercise to exhaustion at 100% of peak ${\dot{V}}_{O_{2}}$ (Kayser, Ferretti et al., 1993) as well as during 45 s supramaximal exercise at a work rate corresponding to 200% of peak ${\dot{V}}_{O_{2}}$ (Grassi et al., 1995).

Also, [La]_b_ at the same submaximal work rate confirmed the presence of the lactate paradox, with higher values, vs. pre‐expedition, after 1 week at altitude, and decreases after 3 and 5 weeks vs. after 1 week at altitude (Grassi et al., 1996).

The lower peak [La]_b_ at altitude was associated with a reduced maximal glycolytic flux, as estimated (with some assumptions) by the maximal rate of [La]_b_ accumulation during supramaximal exercise of increasing duration, performed at 200% of the work rate associated with peak ${\dot{V}}_{O_{2}}$ (Grassi et al., 1995).

Looking for potential causes of the lactate paradox, we turned our attention to acid–base balance. After 1 week at altitude the subjects, as expected, were in a condition of respiratory alkalosis, which was partially compensated at 5 weeks (Grassi et al., 1996). As hypothesized by Cerretelli et al. (1982), the lower peak [La]_b_ in acclimatized subjects could be related to the condition of partially compensated respiratory alkalosis, leading to decreased blood and tissue [HCO_3_^−^] levels, to an impaired buffering capacity, to an enhanced pH drop following H^+^ accumulation, resulting in an inhibition of regulatory enzymes of glycolysis (see also Cerretelli & Samaja, 2003). This hypothesis was not confirmed in another study performed in the Pyramid (Kayser, Ferretti et al., 1993), in which lower peak [La]_b_ values were still observed following the oral administration of sodium bicarbonate (0.3 g/kg of body mass), which restored the alkali reserve to pre‐expedition values.

According to other experiments performed in the Pyramid by Kayser et al. (1994), the lower peak [La]_b_ observed at altitude, during exercise performed with large muscle masses (such as cycle ergometer exercise) could be attributed, at least in part, to a reduced central drive to locomotor muscles, possibly aimed at preventing the failure of vital systemic functions. On the other hand, exercise with small muscle groups (e.g. forearm muscles) would maintain sea‐level capacity to perform maximal work, as well as peak [La]_b_ values.

Overall, in our experiments in the Pyramid Laboratory we confirmed the presence of the lactate paradox during acclimatization to 5050 m, which was not directly related to a reduced muscle mass, to the reduced peak work rate, to the presence of hypoxia itself, or to the reduced buffering capacity. In terms of the general limitations of these studies, no measurements of blood catecholamines were performed, and no muscle biopsies were taken. Overall, if one holds to the premise that lactate^−^ accumulation is directly caused by hypoxia and anaerobic glycolysis, in the search for causes/mechanisms explaining the lactate paradox our Pyramid experiments yielded mainly negative results. The causes/mechanisms remained elusive. As discussed in the following paragraphs, however, for a correct interpretation of the phenomenon the premise mentioned above needs to be revisited. It should also be remembered that, as it happens in several physiological conditions in which different external stimuli are present, and different organs are involved in the responses in an integrated manner, mechanisms of physiological regulation are inevitably redundant (Joyner, 2011). This means that the experimental elimination of one factor, and the subsequent observation of an unchanged response, does not allow to exclude with certainty that the factor is participating in the integrated regulation in the intact system.

## 5 THE NEED FOR A REVISITATION OF THE CONCEPT

The physiological meaning and the mechanistic insights related to the lactate paradox need indeed to be reconsidered, mainly after decades of work by George Brooks on the ‘lactate shuttle’ paradigm (see, for example, the reviews Brooks, 1986, 2009, 2012, 2018, 2023). These studies, together with several others (see below), radically changed our conceptions and perspective about lactate^−^ and ‘anaerobic’ metabolism (see, for example, Ferguson et al., 2018; Gladden, 2004; Poole et al., 2021). In short, the lactate shuttle paradigm and other studies substantially negate [La]_b_ dependence on tissue hypoxia, and see the molecule as a readily combusted fuel that is shuttled from the cytosol of muscle fibres (and other tissues producing it, as a consequence of accelerated glycolysis) to the mitochondria of the same fibre, to other fibres of the same muscle, to other muscles, and to other organs (heart, brain), which utilize lactate^−^ via oxidative phosphorylation or for other purposes, such as glycogen synthesis or gluconeogenesis in liver. In this scenario lactate^−^ would represent a coordinator of whole‐body metabolism and a signalling molecule for various organs and functions, acting as a myokine or exerkine (Brooks, 2023), possibly including epigenetics effects (Wang & Zhu, 2025). Increased [La]_b_ levels (lactate^−^ accumulation in blood) would be the result of a greater lactate^−^ production vs. lactate^−^ utilization. This concept is in agreement with the more ‘classic’ outlook on the issue of di Prampero (1981) and Ferretti & di Prampero (2026) (see below).

In any case, after decades of the lactate shuttle ‘thunderstorm’, two contrasting schools of thought have emerged on the issue of hypoxia and anaerobic glycolysis as the causes for the increased lactate^−^ production and accumulation. The contrasting interpretations were exemplified in a recent debate in the *European Journal of Applied Physiology*. According to Poole & Gaesser (2025) ‘endogenous lactate^−^ accumulation should, perhaps, be considered as a stress response evoked by a certain metabolic strain evoked by intramyocyte perturbations, rather than to an anaerobic process or substantive energy source during heavy‐ or severe‐intensity exercise’. On the other hand, according to Ferretti & di Prampero (2026), [La]_b_ ‘accumulation during exercise at powers above the maximal lactate steady state corresponds to and reflects the metabolic power derived from anaerobic lactic metabolism’.

In terms of the present manuscript the question is: can the two contrasting perspectives outlined above be at least in part reconciled? Moreover, how do the new concepts about lactate^−^ and its metabolism affect the lactate paradox and the physiological inferences deriving from the concept? In the next paragraphs a revisitation of the lactate paradox will be proposed, with the aim of at least in part reconciling the contrasting interpretations presented above. The ultimate aim would be to convince the reader about the relevance and interest of a revisited lactate paradox in the field of integrative and environmental physiology, even after many years following its appearance in the scientific literature.

## 6 BLOOD LACTATE− DURING EXERCISE IN NORMOXIA AND IN ACUTE HYPOXIA

Upon acute exposure to hypoxia, an enhanced sympatho‐adrenergic response to exercise is observed (being part of the stress response), with increased levels of blood adrenaline (Mazzeo et al., 1991, 1994), which appear to be proportional to the hypoxic stimulus. The increased adrenaline levels, occurring also during increasing intensity exercise in normoxia, stimulate glycogenolysis and glycolysis in skeletal muscle fibres (Chasiotis, 1983) as well as blood lactate accumulation (Mazzeo & Marshall, 1989). The accelerated glycolysis inevitably leads to increased lactate^−^ production, because of the near‐equilibrium nature of the lactate dehydrogenase (LDH) reaction, and because LDH has a much higher activity than key regulatory enzymes of glycolytic and oxidative pathways (Ferguson et al., 2018). The increased rate of lactate^−^ production would outstrip the rate of lactate^−^ utilization by oxidative metabolism (in the same fibre, in different fibres, in different muscles, in different organs), thereby leading to increased [La]_b_.

Thus, a critical lack of O_2_, impairing oxidative phosphorylation, does not necessarily need to be present to explain an increased lactate^−^ production during increasing intensity exercise, in normoxia or in acute hypoxia. In other words, ‘anaerobic glycolysis’ and muscle and blood lactate^−^ accumulation would not be directly caused by a critical lack of O_2_, confirming one of George Brooks's main messages (Brooks, 2012). A critical lack of O_2_ impairing oxidative phosphorylation and enhancing anaerobic glycolysis is indeed not present during exercise, even in hypoxia. Intramyocyte $P_{O_{2}}$ levels ($P_{iO_{2}}$) during normoxic or hypoxic exercise substantially decrease vs. those in resting conditions, but are maintained above the critical $P_{iO_{2}}$ (about 2 mmHg) known to be limiting O_2_ utilization by cytochrome *c* oxidase (‘dysoxia’) (see reviews by Clanton et al., 2013; Poole et al., 2021). Richardson et al. (1998) demonstrated that lactate^−^ production is not related to $P_{iO_{2}}$ values (determined by measuring myoglobin saturation by ^1^H nuclear magnetic resonance spectroscopy) but to arterial [adrenaline]. [La]_b_ accumulation would then mainly derive, during increasing intensity exercise in normoxia, and more markedly so during increasing intensity exercise in acute hypoxia, from the accelerated glycolysis and lactate^−^ production driven by various degrees of sympatho‐adrenergic stimulation of glycogenolysis, exceeding lactate^−^ utilization by oxidative metabolism.

## 7 A RECONCILIATION OF TWO CONTRASTING INTERPRETATIONS

A more indirect link between tissue hypoxia and lactate^−^ production is, however, present. Accelerated glycolysis and lactate^−^ production is indeed associated with increasing work rate and with decreasing $P_{iO_{2}}$ levels, even without reaching actual dysoxia, through increases in [ADP]∙[P_i_]/[ATP] (Clanton et al., 2013). Any increase in [ADP]∙[P_i_]/[ATP], required to elevate ${\dot{V}}_{O_{2}}$, represents a very powerful stimulus for glycolytic activity (Wilson, 1994). In other words, an indirect relationship between intracellular hypoxia and lactate^−^ production would exist, even if lack of O_2_ (dysoxia) cannot be considered the direct cause of anaerobic glycolysis and lactate^−^ production.

Accelerated glycolysis leading to excessive lactate^−^ production (vs. utilization), even if not directly caused by anaerobiosis or lack of O_2_, would nonetheless determine ATP production along a metabolic pathway which can be defined ‘anaerobic’ since it does not involve O_2_. Glycolysis leading to lactate^−^ production, therefore, could be termed ‘anaerobic’ not in terms of the cause of the accelerated flow, but in terms of an ‘anaerobic’ energy yield. In this scenario, the classic concept by di Prampero (1981) and by Ferretti & di Prampero (2026), according to which blood lactate^−^ accumulation, deriving from an excess of lactate^−^ production vs. lactate^−^ utilization, represents an ‘anaerobic’ energy yield, would not be in contradiction with Brooks's lactate shuttle.

The increased blood lactate^−^ accumulation for the same submaximal work rate in acute hypoxia, vs. normoxia, would represent an advantage in terms of an enhanced capacity to distribute to muscles and other organs a molecule, lactate^−^, which has a crucial role as substrate for oxidative phosphorylation, for glycogen synthesis and gluconeogenesis, and for signalling purposes. One criticism which could be made to Brooks's scenario is that it somehow ‘scotomizes’ H^+^, which, at the pH of the body, inevitably derives from the dissociation of lactic acid (the terminal product of glycolysis) into H^+^ and lactate^−^, with the obvious negative effects on pH homeostasis (Poole et al., 2021; Wasserman et al., 1973). The disadvantage represented by the increased [H^+^] levels, inevitably associated with lactate^−^ accumulation, would be attenuated or eliminated, at low work rates, by bicarbonate (HCO_3_^−^) buffering. Metabolic acidosis would on the other hand develop at higher work rates. The pH perturbation would be more pronounced in chronic hypoxia, as a consequence the of the decreased HCO_3_^−^ levels attributable to the renal compensation (increased HCO_3_^−^ elimination with urine) of respiratory alkalosis (Cerretelli & Samaja, 2003; Cerretelli et al., 1982).

## 8 CHANGES OCCURRING DURING ALTITUDE ACCLIMATIZATION

In chronic hypoxia (i.e. with altitude acclimatization), the scenario changes, at least in part, following the intervention of a series of mechanisms which would ultimately result in decreased [La]_b_ for the same submaximal work rate and in decreased peak [La]_b_, that is the lactate paradox.

To begin with, the sympatho‐adrenergic stimulation would be attenuated during altitude acclimatization, compared to acute altitude exposure, as demonstrated by the arterial adrenaline levels observed in subjects exposed to 4300 m (Mazzeo et al., 1991, 1994). After considering the role of the sympatho‐adrenergic stimulation in the regulation of glycogenolysis, glycolytic flux, lactate^−^ production and accumulation (see above), the decreased sympatho‐adrenergic stimulation following acclimatization would go in the direction of decreasing lactate^−^ production by skeletal muscle fibres and lactate^−^ accumulation in blood.

It should be noted, however, that unchanged arterial catecholamine levels were observed by van Hall et al. (2009) in subjects after 8 weeks vs. after 2 weeks of exposure at 4100 m. Both at 2 weeks and at 8 weeks the levels were higher than those obtained in acute normobaric hypoxia induced at sea level. No clear explanation can be given for these contrasting observations, in terms of the sympatho‐adrenergic response during acclimatization. A potential confounding factor is that van Hall et al. (2009), differently from Mazzeo et al. (1991, 1994), did not perform measurements early during altitude exposure. According to Hansen & Sander (2003) a sympathetic neural overactivity (determined directly by peroneal microneurography) was present after 31 days at 5260 m vs. sea level values. Also in this case, however, no measurements were performed early after altitude exposure, so that the pattern of the variable *during* acclimatization could not be determined.

A series of adaptive systemic phenomena take place during acclimatization, aimed at preserving convective O_2_ delivery to tissues. A sustained hyperventilation is observed, which decreases the difference between inspiratory $P_{O_{2}}$ and alveolar $P_{O_{2}}$. Increased haematocrit (Ht) and haemoglobin concentration ([Hb]) in blood are also described following hypoxia‐inducible factors (HIF) and erythropoietin responses (Semenza, 2012). During the first couple of weeks of altitude acclimatization, the increased [Hb] would be mainly related to a decreased plasma volume, whereas during the ensuing weeks an increase in red blood cell volume would occur (Siebenmann et al., 2017). The increased [Hb] and decreased blood volume maintain arterial O_2_ concentration ($C_{aO_{2}}$) at values not different from those at sea level, up to an altitude of about 7000 m (Grocott et al., 2009). Peak cardiac output decreases as a function of altitude, and following $C_{aO_{2}}$ restoration by the increased [Hb] also cardiac output for the same submaximal work rate returns to sea level values, after a transient increase occurring in acute hypoxia (Reeves et al., 1992).

According to extensive work carried out during the last couple of decades, however, the [Hb] increase during altitude acclimatization (not seen in high‐altitude populations such as Tibetans and Ethiopians; see Wagner, 2022; Wagner & Simonson, 2026) would represent a maladaptive phenomenon, first of all by increasing cardiac work and by impairing cardiac output and microvascular O_2_ delivery (Wagner, 2022), as a consequence of the increased blood viscosity.

Moreover, the increased [Hb] observed with acclimatization does not affect O_2_ partial pressures (Wagner & Simonson, 2026), which remain significantly lower vs. sea level conditions, independently from the time spent at altitude. This inevitably impairs O_2_ diffusion. Moreover, as demonstrated by Piiper & Scheid (1981), diffusive loading of O_2_ from the alveoli to pulmonary capillaries, as well as diffusive unloading of O_2_ from microvascular Hb to tissue mitochondria, depend quantitatively on the ratio between diffusive O_2_ conductance (numerator) to perfusive O_2_ conductance (denominator). Thus, in the presence of a decreased diffusive O_2_ conductance (partially compensated for by the left‐shifted Hb‐O_2_ dissociation curve [Winslow et al., 1984] and by the increased mean slope of the curve), the increased perfusive O_2_ conductance, deriving at the peripheral level from the increased [Hb] and $C_{aO_{2}}$, would significantly impair gas exchange. A similar situation, in terms of diffusive and perfusive conductances, would occur at the alveolar‐capillary level.

In terms of peripheral O_2_ diffusion, the number of capillaries and capillary density are unchanged with acclimatization (Cerretelli & Hoppeler, 1996; Lundby et al., 2004), with the exception of cases of muscle fibre atrophy described following prolonged sojourns at extreme altitudes (Cerretelli & Hoppeler, 1996; Hoppeler et al., 1990). Myoglobin concentration does not seem to increase, at least in humans (Murray & Horscroft, 2016). No significant changes in the distribution of muscle fibre types are observed (Cerretelli & Hoppeler, 1996).

Although the net result of the changes occurring during altitude acclimatization, briefly discussed above, is difficult to determine, O_2_ transport from ambient air to mitochondria in skeletal muscle is significantly impaired in chronic hypoxia, in an altitude‐dependent manner, and the muscles are hypoxic (Ward et al., 2000). During acclimatization a series of cellular responses to hypoxia occur, orchestrated by the HIF family of transcription factors (Semenza, 2012). These adaptations are nicely summarized in the reviews by Cerretelli & Hoppeler (1996), Horscroft & Murray (2014), Murray & Horscroft (2016) and Murray et al. (2018). A regulated metabolic remodelling occurs within muscle fibres, mainly aimed at attenuating the difference between O_2_ delivery and O_2_ demand. In other words, in the presence of a substantial decrease in O_2_ delivery, compensatory mechanisms are established with the aim to reduce O_2_ demand.

A decreased oxidative capacity is observed (Cerretelli & Hoppeler, 1996), which is manifested metabolically even before a reduced mitochondrial mass occurs (particularly of subsarcolemmal mitochondria) following prolonged exposure to extreme altitudes (Cerretelli & Hoppeler, 1996; Hoppeler et al., 1990). A downregulation of oxidative phosphorylation, tricarboxylic acid cycle and electron transport chain activities occurs (Murray & Horscroft, 2016; Murray et al., 2018), as manifested by a reduced respiratory capacity determined by high‐resolution respirometry (HRR) in subjects exposed to 3454 m in the Swiss Alps (Jacobs et al., 2013). A 30% decrease of OXPHOS capacity was described by the same group, after 28 days at 3454 m, after this variable was normalized to citrate synthase activity (estimate of mitochondrial mass) (Jacobs et al., 2012). The picture becomes even more interesting when OXPHOS capacity is normalized to mitochondrial cristae density. Mitochondrial cristae are infoldings of the inner mitochondrial membrane, in which most complexes for energy transduction are localized. The increased respiration after OXPHOS capacity is normalized to total fibre cristae density (Schytz et al., 2025) suggests an increased efficiency of mitochondrial respiration.

A decreased fatty acids oxidation and an enhanced glucose utilization increase the efficiency of oxidative phosphorylation (Murray & Horscroft, 2016); an increased respiratory coupling is also described (decreased LEAK respiration by HRR; Jacobs et al., 2013), as well as downregulation of uncoupling protein 3. Lower levels of peroxisome proliferator‐activated receptor γ coactivator 1‐α (PGC‐1α) decrease mitochondrial biogenesis (Murray & Horscroft, 2016), in association with a decreased protein synthesis, which may ultimately lead to muscle atrophy. The decreased oxidative capacity is also aimed at attenuating mitochondrial reactive oxygen species production, which is known to be increased in hypoxic conditions (Murray & Horscroft, 2016).

In the presence of a downregulation of oxidative metabolism, one might expect an enhanced ‘anaerobic glycolysis’. Evidence in this direction, however, in humans is limited (Horscroft & Murray, 2014), and available data point to an unchanged maximal activity of glycolytic enzymes (Cerretelli & Hoppeler, 1996). In a paper which is one of the last contributions of Paolo Cerretelli to the field of altitude physiology, a decreased activity of glycolytic enzymes in subjects acclimatized to altitude was described (Levett et al., 2015). As pointed out by Cerretelli & Hoppeler (1996), the maximal potential activity of glycolytic enzymes, determined in vitro in muscle fibres obtained by biopsy, cannot allow direct inferences on the maximal metabolic flux in vivo, which could be affected by upstream inhibition and/or by changes in the velocity constant of rate‐limiting enzymes.

In any case, the cardinal features of the lactate paradox (lower [La]_b_ for the same submaximal work rate, lower peak [La]_b_) point to an attenuated blood lactate^−^ accumulation with acclimatization. The decreased sympatho‐adrenergic stimulation, a less pronounced intracellular hypoxia, mainly deriving from the reduced O_2_ demand, and resulting in an attenuation of the [ADP]∙[P_i_]/[ATP] increase (Clanton et al., 2013), would go in the direction of a slowed rate of glycolysis and less lactate^−^ production and accumulation. At the end, a ‘tighter coupling’ between lactate^−^ production and lactate^−^ utilization by oxidative metabolism (characterized by a reduced O_2_ demand in response to a reduced O_2_ transport, see above) would occur, leading to the ‘lactate paradox’, as originally proposed by Peter Hochachka (1988), Bengt Kayser (1996) and Peter Wagner (Wagner & Lundby, 2007).

## 9 A REMARKABLE SERIES OF METABOLIC ADJUSTMENTS

In this scenario, and after taking into consideration Brooks's paradigm of the lactate shuttle and other recent concepts on lactate metabolism, muscle and blood lactate^−^ accumulation during increasing intensity exercise would reflect an intrinsic mismatch between a faster ‘speed’ of glycolysis and a relatively slower speed of oxidative phosphorylation, possibly aimed at accumulating lactate^−^ in blood, to be distributed for different purposes throughout the body. All these phenomena would be indirectly related to (although not directly caused by) intracellular hypoxia, and lactate^−^ accumulation would represent an ‘anaerobic’ (i.e. occurring without O_2_ intervention) energy yield. Greater blood lactate^−^ accumulation would derive from a greater lactate^−^ production vs. lactate^−^ utilization. Acute hypoxia would further accelerate glycolysis through an enhanced sympatho‐adrenergic stimulation, would worsen intracellular hypoxia, and would increase [ADP]∙[P_i_]/[ATP], with the resulting effect of a greater [La]_b_ for the same absolute work rate. During altitude acclimatization the sympatho‐adrenergic stimulation would fall, intracellular hypoxia would be attenuated (mainly through a reduced O_2_ demand), the [ADP]∙[P_i_]/[ATP] increase would subside, that is, a tighter coupling between lactate^−^ production and utilization would ensue, with the end result of decreased [La]_b_ for the same absolute work rate and at peak work rate.

In other words, a complex and remarkable series of metabolic adjustments would be involved, with no need to invoke a ‘paradox’, although several mechanisms still need to be clarified. The author is well aware that several of the proposed mechanisms are only indirectly demonstrated, and would need a more direct experimental confirmation, and that some conflicting results are present. In more general terms, however, the revisited lactate paradox should represent another example of the scientific interest which can still be raised by ‘old fashioned’ integrative and environmental physiology (Joyner, 2011; Wagner & Paterson, 2011; Grassi et al., 2019).

## AUTHOR CONTRIBUTIONS

Sole author.

## CONFLICT OF INTEREST

None declared.

## FUNDING INFORMATION

None.

## GENERATIVE AI STATEMENT

No part of the content of this article or results presented were generated by a large language model or other form of generative artificial intelligence.

## Data Availability

The datasets used and analysed during the current study is available from the corresponding author on reasonable request.
